# The Contribution of Machine Learning in the Validation of Commercial Wearable Sensors for Gait Monitoring in Patients: A Systematic Review

**DOI:** 10.3390/s21144808

**Published:** 2021-07-14

**Authors:** Théo Jourdan, Noëlie Debs, Carole Frindel

**Affiliations:** 1 Univ Lyon, INSA Lyon, Inria, CITI, F-69621 Villeurbanne, France; theo.jourdan@creatis.insa-lyon.fr; 2 Univ Lyon, INSA-Lyon, Université Claude Bernard Lyon 1, UJM-Saint Etienne, CNRS, Inserm, CREATIS UMR 5220, U1294, F-69621 Villeurbanne, France; noelie.debs@creatis.insa-lyon.fr

**Keywords:** gait, chronic pathology, tracker, wearable, validation, gold standard, machine learning, systematic review

## Abstract

Gait, balance, and coordination are important in the development of chronic disease, but the ability to accurately assess these in the daily lives of patients may be limited by traditional biased assessment tools. Wearable sensors offer the possibility of minimizing the main limitations of traditional assessment tools by generating quantitative data on a regular basis, which can greatly improve the home monitoring of patients. However, these commercial sensors must be validated in this context with rigorous validation methods. This scoping review summarizes the state-of-the-art between 2010 and 2020 in terms of the use of commercial wearable devices for gait monitoring in patients. For this specific period, 10 databases were searched and 564 records were retrieved from the associated search. This scoping review included 70 studies investigating one or more wearable sensors used to automatically track patient gait in the field. The majority of studies (95%) utilized accelerometers either by itself (N = 17 of 70) or embedded into a device (N = 57 of 70) and/or gyroscopes (51%) to automatically monitor gait via wearable sensors. All of the studies (N = 70) used one or more validation methods in which “ground truth” data were reported. Regarding the validation of wearable sensors, studies using machine learning have become more numerous since 2010, at 17% of included studies. This scoping review highlights the current state of the ability of commercial sensors to enhance traditional methods of gait assessment by passively monitoring gait in daily life, over long periods of time, and with minimal user interaction. Considering our review of the last 10 years in this field, machine learning approaches are algorithms to be considered for the future. These are in fact data-based approaches which, as long as the data collected are numerous, annotated, and representative, allow for the training of an effective model. In this context, commercial wearable sensors allowing for increased data collection and good patient adherence through efforts of miniaturization, energy consumption, and comfort will contribute to its future success.

## 1. Introduction

Human gait assessments study human movement and aim to quantify gait characteristics with various spatiotemporal parameters, such as stride speed and length, step length, cadence, standing, double support, and swing times [1]. Normal gait corresponds to an individual’s motion pattern, and deviation in gait from this normal pattern can indicate a change in health status. In this regard, recent works have demonstrated that gait could have a link to functional health and could be an indicator for the course of chronic disease and, hence, rehabilitation feedback [2]. For example, ref. [3] demonstrated the value of studying gait asymmetry in post-stroke patients, ref. [4] identified gait variability as a marker of balance in Parkinson’s disease, and ref. [5] described changes in gait and balance in the elderly. As a result, there is a move towards using gait analysis to aid in patient health assessment and monitoring.

Traditional methods for gait analysis in patients typically use walk tests as a standard assessment [6,7]. A walk test is an examination carried out over a fixed duration and/or distance in order to easily access speed measurements. The most commonly used walk test is the six-minute walk test (6MWT) [8], which assesses endurance at a comfortable speed for the subject by measuring the distance walked in 6 min along a straight corridor. Even though these tests are widely used to establish a link between the gait and physical state of the patient, important long-term gait longitudinal patterns or transition patterns from one daily activity to another are not measured and cannot be explored. The ability to explore these patterns, such as the transition from turning to sitting [9], frequency of falls [10], or freezing episodes [11] is important because recent literature suggests that they may be able to inform about a deterioration in the patient’s state of health and, therefore, of their chronic condition.

Emerging technologies offer the possibility to improve the evaluation of traditional methods by increasing the quality and the duration of the window of data acquisition by measuring gait in daily activities over long periods of time. Wearable devices with embedded sensors allow in particular for the passive collection of various data sets, which can then be used to develop algorithms to assess gait in real life conditions and over long periods of time [12,13]. This opens up many perspectives, especially in the case of chronic diseases where the disease profile varies for each individual and has fluctuating symptoms. Twenty-four hour home monitoring in a real environment is an ideal solution for an accurate diagnosis of symptoms as well as good patient compliance [14].

In the past decade, commercial wearable sensors have been used not only in the consumer market but also in research studies. In particular, wearable sensors are used in physical activity monitoring for measurements and goal setting [15]. More recently, a more specific use of these sensors was introduced in research studies in medicine and rehabilitation [16,17]. Wearable sensors for gait assessment have been primarily conducted in a lab and with controlled protocols [18], traducing that commercial sensors can be challenging to deploy and validate. More recently, the testing of the sensors in patient monitoring has expanded into real-life conditions. Previous research has shown significant differences in spatiotemporal gait parameters between similar in-lab and in-field studies [19], illustrating the importance of establishing commercial sensor validity for long-term patient monitoring and for detecting events and more particularly deviations from normal human gait.

There are already many reviews on the validation of commercial wearable sensors available in the literature, and most were interested in monitoring activity on healthy subjects [15,20,21,22] while others have taken a descriptive approach centered on a very specific medical application [18,23,24]. However, few studies focus on the validation methods, the ground truth used, and how the reference data are annotated. A common validation method is to use inferential statistics, such as a regression analysis to explore and model the relationship between sensor and ground truth data. These approaches typically assume that the relationship between sensor and ground truth data follows a linear pattern. Linear regression has the advantage of being simple to use and to interpret. In comparison with these linear methods, the nonlinear methods fit more types of data in terms of shape and are hence recognized as being more general. Some nonlinear approaches such as machine learning have the advantage of being less dependent on the assumption of the model and very recently produced promising results in sensor validation [25,26]. Nonlinearity seems particularly interesting in terms of patient monitoring in order to integrate networks of several sensors placed at different places on the patient [27,28] and for high-level tasks (such as the classification of patients into groups according to the evolution of a disease) [29,30], which requires the integration of various information on locomotion and control systems involved in complex gait regulation [31,32].

In this paper, our aim was to conduct a systematic review (i) to determine the statistical methods currently used for the validation of sensors and (ii) to determine to what extent machine learning (ML) is used as a statistical method for this validation step.

## 2. Methods

This scoping review is reported using the Preferred Reporting Items for Systematic reviews and Meta-Analyses Extension for Scoping Reviews (PRISMA-ScR) checklist [33].

### 2.1. Databases

We conducted a literature search of the PubMed, SCOPUS, ScienceDirect, Web of Science, IEEE Xplore, ACM Digital Library, Collection of Computer Science Bibliographies, Cochrane Library, DBLB, and Google Scholar (first 50 results) databases for all literature published between 2010 and 2020.

### 2.2. Literature Search

The literature search strategy included a combination of keywords to identify articles that addressed (i) gait assessment/detection, (ii) wearable and connected technology, (iii) chronic pathology monitoring, and (iv) validation. Keywords included “gait”, “walk”, “actigraphy”, “actimetry”; “smartphone”, “wearable”, “mobile device”, “IoT”; “chronic disease”, “rehabilitation”, “medicine”; “validity”, “validation”, “reliability”, and “reproductibility”. The full search term strategy that was used for each database is given in Table A1 of Appendix A.

### 2.3. Inclusion Criteria

Only peer-reviewed journals or conference papers were included in this review if they were published between January 2010 and December 2020 and were written in English. In addition, eligible articles had to complete all of the following criteria as part of the content given in the article:

1. The study must be centered on gait or posture analysis (e.g., detect stance and swing phases, detect the risk of falling, etc.). Studies focusing only on activities or step counting were excluded.
2. Given the application to remote monitoring in patients, only devices allowing wireless data flow wer considered. This flow had to have been conducted using bluetooth between the device and the smartphone to then send data by Wi-Fi to a remote server. Sensors that temporarily store the data locally and send the data a posteriori when a Wi-Fi connection is available were also included.
3. The devices had to have been used in a clinical setting for long-term follow-up or rehabilitation of a chronic pathology. Studies on young or healthy patients and on animals were excluded.
4. The validity of the sensor and the resulting indicators must have been assessed. Therefore, a ground truth must be proposed and the study must include at least one statistical measure (e.g., statistical test, correlation, and mean square error) or one evaluation metric (e.g., accuracy, F1-score, precision, and sensitivity) to indicate the performance of the sensor on detecting the associated gait feature.

Review articles, commentary articles, study protocol articles, and any other articles without reported results from empirical research were excluded.

### 2.4. Selection of Articles

**Figure 1:**
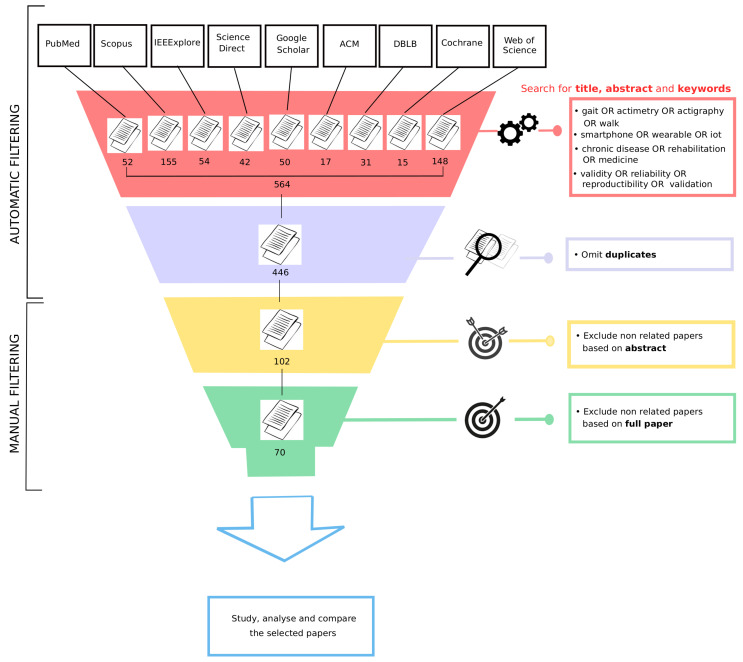
Diagram of the article-selection process.

The records retrieved from the databases were gathered in CSV files. All duplicate articles were removed. First, we reviewed the titles and abstracts of all articles (Figure 1). During this first phase of selection, articles were excluded if they did not describe at least one wearable device used to automatically assess gait as part of the follow-up of a chronic pathology, with particular attention paid to the validation of the device. If this information could not be verified from the title and/or abstract, the article’s full text was reviewed in a further screening phase to determine whether it fit the eligibility criteria. Moreover, if the abstract indicated that the study was not peer-reviewed, was not written in English, was not accessible online, or corresponded to a study conducted on animals, it was excluded. After the initial title/abstract selection process, we evaluated the full text of the remaining articles. Articles were then excluded if they did not meet the eligibility criteria (Figure 1).

### 2.5. Data Extraction

Three research assistants independently extracted the following study characteristics from the final set of eligible studies using a custom-made data extraction worksheet. Here are the different characteristics identified for the analysis of identified papers in the context of our systematic review:

1. Sample size: the total number of participants for each study.
2. Pathology: the disease monitored in the study.
3. Duration of data collection: how long the participants wore the sensor(s) to collect data for the study.
4. Condition of data collection: specifies on whether the study was conducted in a laboratory or in free-living conditions.
5. Number of wearable devices: the total number of wearable devices in which the sensor’s signal data were used to study the patient’s gait. Any other equipment that was part of the acquisition system but did not provide data to evaluate the gait was not included in this count.
6. Type of sensor(s): the type of sensor embedded within the wearable device(s) used to assess gait.
7. Device brand(s) and model(s): the specific brand and model of the wearable device(s) used in the study.
8. Location of device(s): details specific to the placement/location of wearable device(s) on the patient’s body.
9. Gait indicators measured by the device(s): gait outcomes that were derived from the signal recorded on the device. In some studies, several gait indicators were extracted from the raw data.
10. Ground-truth method(s): the method that was used in the study to evaluate the performance of the device(s) to assess gait.
11. Evaluation metric(s) of the device(s): any evaluation metric, reported either in the text, a figure, or a table, that described the performance of the wearable device(s) on assessing gait. Only evaluation metrics that were exclusively used to study gait were included.

### 2.6. Summarizing Data and Categories

Mean and standard deviation were calculated for each extracted numerical variable (sample size, duration of data collection, and number of devices). Frequency tables were constructed for each extracted categorical variable (pathology, condition of data collection, sensor types, device brand and model, device location, ground-truth methods, gait features, and evaluation metrics). Regarding these categorical variables, here are the categories that we considered and their meanings. These categories are not exhaustive of all possible types of categories but correspond to those proposed in the context of the included studies.

The devices are categorized according to three types: (i) *smartphone*, (ii) *inertial measurement unit* (IMU), and (iii) *single sensor*.

The device location is categorized according to four levels: (i) *superior*, if the device was carried in the hands or on the arms; (ii) *inferior*, if the device was carried on the legs or feet; (iii) *chest*, if the device was carried on the chest or the trunk; and (iv) *free location*, if the device was in a pocket or more prone to moving around, or if the its location on the body was not distinguished.

The ground-truth methods are categorized according to six levels: (i) *controls*, where a group of subjects served as a reference; (ii) *expert*, where the data were analyzed with regard to annotations made by experts; (iii) *med device*, where the data were analyzed with regard to a portable device already used in clinical routine; (iv) *medical*, where the data were analyzed with regard to a medical examination/test or clinical score; (v) *metrologic*, where other high resolution equipment were used as a reference; and (vi) *user annotations*, where the data were analyzed with regard to annotations made by patients during the use of the device.

The gait features are categorized according to three levels: (i) *low*, where the analysis was conducted on raw signals without postprocessing; (ii) *medium*, where the analysis was based on statistical descriptors extracted from the signals (mainly statistical moments or common signal processing features); and (iii) *high*, where the analysis was based on descriptors at a high level of representation that disregards the technical characteristics of the equipment or methods used (e.g., step length, cadence, and number of steps).

Finally, the evaluation methods are categorized according to five levels: (i) *descriptive stat*, where evaluation was carried out through descriptive statistics only; (ii) *descriptive stat + test*, where evaluation was carried out through descriptive statistics with statistical tests; (iii) *linear models + stat test*, where evaluation was carried out through linear models with statistical tests; (iv) *machine learning*, where evaluation was carried out through machine learning only; and (v) *machine learning + stat test*, where evaluation was carried out through machine learning with statistical tests.

## 3. Results

In this section, we analyze the selected papers by categorizing them following different criteria in order to extract common patterns and trends.

### 3.1. Literature Search

Figure 1 details the entire process of paper selection for this review. The literature search (made from the queries given in Table A1 of Appendix A) produced 564 research articles, with 118 duplicates, resulting in 446 articles to be screened. After an initial screening, which consisted of reviewing all article titles and abstracts, the full content of 102 of these articles was screened in more detail for eligibility. After removing the articles that did not meet the inclusion criteria detailed in Section 2.3, 70 articles were deemed eligible for the review [34,35,36,37,38,39,40,41,42,43,44,45,46,47,48,49,50,51,52,53,54,55,56,57,58,59,60,61,62,63,64,65,66,67,68,69,70,71,72,73,74,75,76,77,78,79,80,81,82,83,84,85,86,87,88,89,90,91,92,93,94,95,96,97,98,99,100,101,102,103].

**Figure 2:**
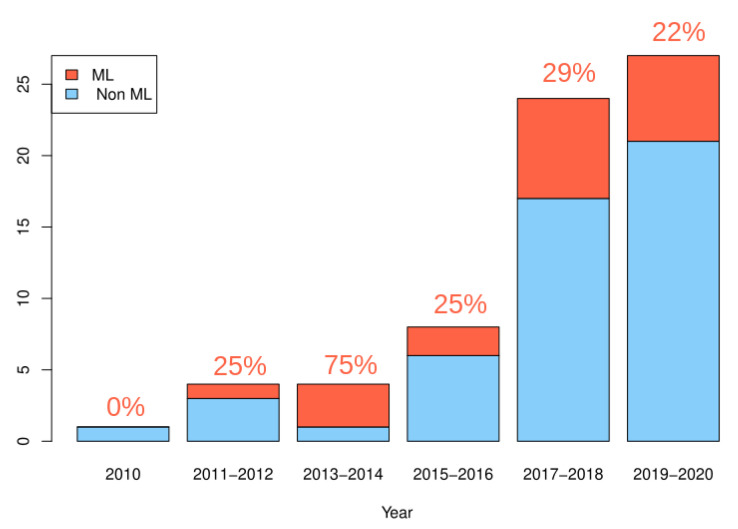
Evolution of the number of papers considering the issue of validation for the use of commercial wearable devices in chronic disease monitoring, with a distinction between papers using machine learning (in red) or not (in blue). The percentages given in red represent the proportion of studies using machine learning.

The number of studies related to the issue of validation on sensors used for patient monitoring has significantly increased since 2010, with a number of papers between 2017 and 2020, more than twice the number of papers between 2010 and 2017 (see Figure 2). Studies using machine learning as a validation method also became more numerous since 2010 [34,35,36,38,45,53,60,63,68,69,70,77,79,80,81,86,95,97], with a stable proportion compared to the total number of studies per year.

### 3.2. Clinical Context

**Figure 3:**
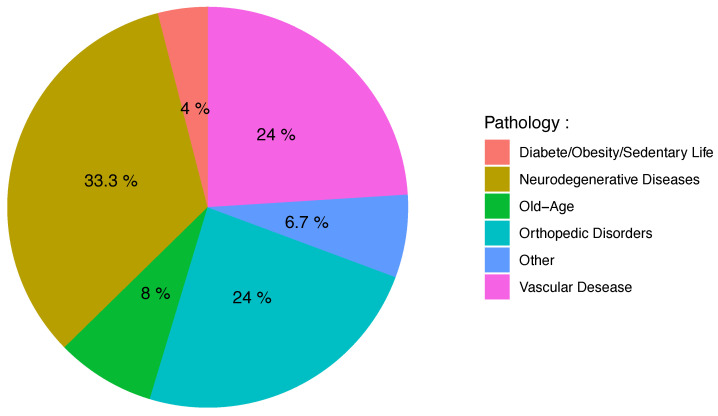
Pie chart representing the frequency of pathology types in included studies.

The sample size of the studies ranged from 1 to 130 participants, with a mean of 37.89 participants (SD = 30.68) per study. The duration of data collection in two different conditions (laboratory or free living) varied and was not always reported with an exact numerical value or unit. Therefore, in Table 1, we only report the ranges of acquisition times that go from hours to years. Among the selected studies, as displayed in Figure 3, 33% (N = 25) focused on neurodegenerative diseases [35,36,37,39,44,50,54,55,57,58,60,61,63,70,72,77,79,80,81,86,90,92,94,98,103], 24% (N = 18) focused on orthopedic disorders [34,47,52,59,65,71,73,75,76,78,83,85,89,91,96,97,99,101], 24% (N = 18) focused on diseases of vascular origin [40,43,45,48,49,51,52,53,62,64,67,68,69,87,91,95,99,102], 8% (N = 6) focused on aging and associated pathologies [38,56,66,88,91,100], and 4% (N = 3) focused on diseases associated with poor lifestyle [42,62,74]. Finally, five studies were classified as “others” [41,46,82,84,93] because they could not be grouped together in an existing group.

### 3.3. Wearable Sensor Types

As detailed in Table 2, the most frequently used type of wearable device is the Inertial Measurement Unit (IMU; N = 39) [34,37,44,46,52,54,55,56,57,58,60,61,62,63,66,71,72,73,75,78,79,81,82,83,84,87,88,90,91,92,93,94,95,97,98,99,100,101,102], and then, almost equally, the smartphone (N = 18) [38,39,40,41,42,43,45,47,51,64,68,69,70,76,77,86,89,103] and a single sensor (N = 17) [35,36,38,40,48,49,50,53,59,65,67,69,74,80,85,96,103]. The majority of studies (N = 56) [34,35,36,37,38,40,43,44,48,49,51,52,53,54,55,56,57,58,60,61,62,63,65,66,67,69,70,71,72,73,74,75,77,78,79,80,81,82,83,84,85,87,88,89,90,91,92,93,95,97,98,99,100,101,102,103] used multi-sensor systems (incorporating more than one sensor) to automatically assess gait in chronic pathologies. On average, 5.78 wearable sensors (SD = 8.43) were used in the studies, with a range of 1 to 64 sensors (see Table 2). As depicted in Table 3, the most commonly utilized sensor was an accelerometer (95%) either by itself (N = 17) or embedded into a device (N = 57). The second most frequently used sensor was a gyroscope (51%) followed by magnetometer (14%) and others (16%).

**Figure 4:**
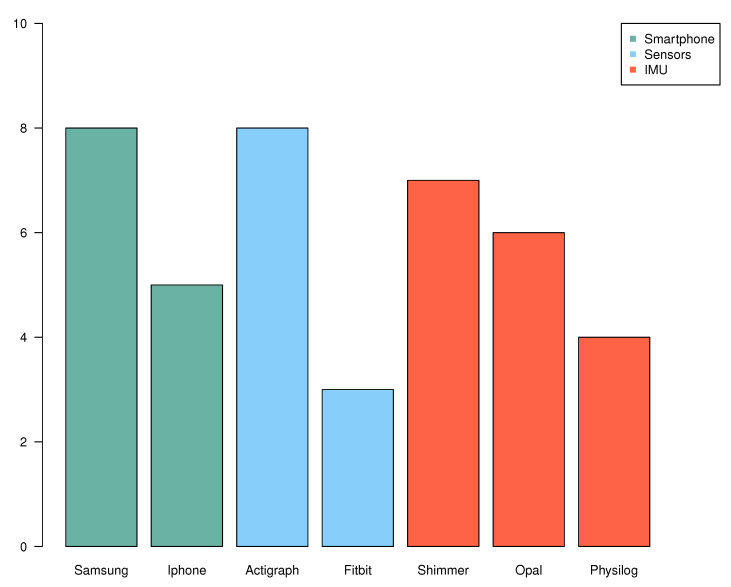
Frequencies of the most used brands (number of occurrences > 3) by type of device (smartphone, sensor, and IMU). Among smartphones, seven papers used Samsung and five used iPhone (bars in green). Among sensors, eight papers used Actigraph and three used Fitbit (bars in blue). Finally, among IMUs, seven papers used Shimmer, six papers used Opal, and four used Physiolog (bars in red).

Figure 4 reports the different brands used for smartphones, sensors, and IMUs. Regarding smartphones, Samsung [41,45,51,68,69,77,86,103] and iPhone [40,42,69,76,89] are the most represented, certainly because of their health applications made for gait recording. Actigraph is the most commonly used brand for sensors [38,40,48,49,67,71,74,85,96,103]. Regarding the different brands in IMU, there is no particular brand that stands out.

### 3.4. Data-Acquisition Conditions

Most of the papers collected their data in laboratory conditions (N = 53) [34,35,36,37,38,39,40,41,42,43,44,45,47,48,49,54,56,57,58,60,61,63,64,65,67,68,69,70,71,72,73,75,76,78,79,80,81,82,83,84,85,87,88,89,90,91,92,95,97,99,100,101,102], while a smaller part collected data in free living conditions (N = 17) [46,50,51,59,77,85,86,96,103] (see Table 1).

Regarding the positioning of sensors and/or devices (Table 4), 60% of the studies placed them on an inferior part of the body [35,36,37,40,47,48,49,52,53,54,55,56,57,58,60,62,63,67,70,71,73,74,75,76,78,79,80,81,83,85,87,88,90,91,92,95,96,97,98,99,100,102,103], generally on the feet (N = 14) or on the hips (N = 6). The chest was also widely used (49%) [34,37,38,39,44,48,50,54,55,56,59,60,61,64,65,67,70,72,73,75,77,79,83,84,89,90,92,93,94,95,97,99,101,102]; 17% of the studies carried out sensor positioning on the hands and arms [38,40,46,48,52,63,66,67,77,80,82,90,102], while the other 17% used a trouser or jacket pocket [42,43,45,50,51,59,68,70,77,86,89,103]

### 3.5. Gait Indicators

The majority (70%) of studies (see Table 5) used high-level features for gait analysis [35,36,37,39,40,43,44,45,46,48,49,50,51,54,55,56,57,58,59,62,65,66,67,71,72,74,75,76,77,78,82,83,84,85,86,87,88,89,90,91,92,93,94,95,96,97,99,102], which can be correlated to the high use of smartphones (in the studies reviewed; see Table 3) that already compute this type of features on the device.

A significative part of the studies (28%) used medium-level features [34,38,42,45,47,52,53,59,61,63,64,68,69,70,73,79,98,101,103], while low-level features (raw data) are much less exploited (8%) [41,60,61,80,81,100].

### 3.6. Ground Truth

**Figure 5:**
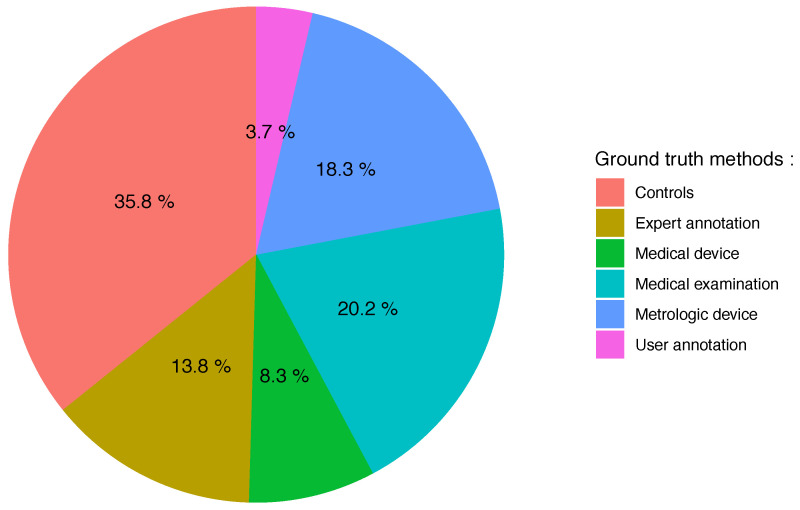
Pie chart representing the frequency of different ground-truth methods identified among the 70 selected papers. These different levels correspond to the categories described in Section 2.6.

To evaluate the validity of commercial wearable sensors for gait monitoring in patients, all of the studies (N = 70) used one or more validation methods in which the “ground truth” data were reported. As illustrated in Figure 5, about half of the studies (53.3%) use annotations and the other half (46.7%) use a reference to validate the results from the sensors. Regarding annotations, most studies use labeling according to two or more groups of subjects (the vast majority of the time, a group of patients and healthy controls) [35,36,37,38,39,41,44,46,47,50,51,53,54,56,58,59,64,66,71,75,76,77,78,79,81,83,84,85,86,90,91,92,93,94,96,97,101,102,103], others use annotations made by experts on data from videos or measurements during the experiment [37,38,40,43,48,52,55,63,67,70,74,80,94,98,100], and four studies [55,64,92,93] had participants self-report via a log or diary. With regard to the reference with which the studies compare the data from the sensors, it concerns a metrological device (18.3%) [35,36,39,41,49,53,54,57,60,61,65,67,72,78,79,83,87,96,97,99] or a medical examination (20.2%) [34,36,39,44,45,50,51,58,59,68,72,73,75,77,81,84,85,90,95,96,102,103] in equal parts and, to a lesser extent (8.3%), a third-party portable medical device [40,42,45,49,62,69,82,89,103].

### 3.7. Evaluation Methods and Metrics

**Figure 6:**
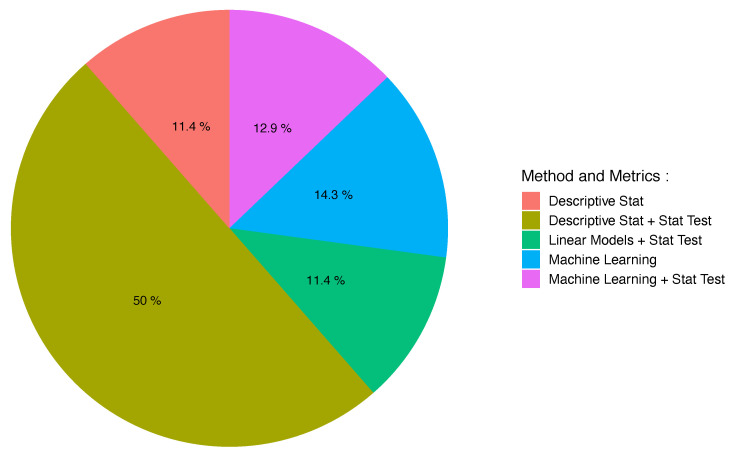
Pie chart representing the percentage of papers using different levels of evaluation identified among the 70 selected papers. These different levels correspond to the categories described in Section 2.6.

The studies often reported multiple and varied evaluation metrics. All reported evaluation outcomes and their corresponding evaluation method are included in Table 6 and depicted in Figure 6. The most common evaluation method was descriptive statistics (61.4%) including or not statistical tests [37,39,40,41,44,46,48,49,51,54,55,58,59,61,62,65,66,67,71,72,74,76,78,82,83,84,85,87,88,89,90,91,92,94,98,99,101,102,103] where correlations, mean errors, or p-values are most commonly reported. The other evaluation methods present models either as a linear model (11.4%) [42,50,52,56,57,73,75,93,96,100] or as a machine learning model (17.2%) [34,35,36,38,45,53,60,63,68,69,70,77,79,80,81,86,95,97]. Due to the lack of a standardized evaluation metric across studies, we do not summarize (calculate mean, standard deviation, etc.) the reported metrics. However, evaluation metric values—as given in the abstract or the conclusion of the associated studies—are available in Table 6.

A closer look at the studies using ML highlights that machine learning-based approaches are often used for high-level validation tasks (see Table 7), such as distinguishing between different groups of patients or stages of disease progression [34,35,36,45,68,70,80,86,97]. This is an important point because ML aims to generalize a model to patients not included in the initial data set. Another point to emphasize, as illustrated in Table 8, is that studies using machine learning as a validation method incorporate a large number of variables (the complete raw signal or a collection of different sensors) [34,60,63,70,77,80,81]. This is not the case in studies using statistical methods that work with a few dozen variables at the maximum and often in a uni-variate way two by two [37,56,59,90,102,103].

### 3.8. Summary of Key Findings

This scoping review included 70 studies related to the validation of commercial wearable sensors to automatically monitor gait in patients published between 2010 and 2020. The majority of studies (95%) used accelerometers either by itself (N = 17 of 70) or embedded into a device (N = 57 of 70), and/or gyroscopes (51%) to automatically monitor gait via wearable sensors. Labeling according to two groups (group of patients and healthy controls) was the most frequently used method (N = 39 of 70) for annotating ground-truth gait data, followed by annotations made by experts on data from videos or measurements during the experiment (N = 15 of 70) and patient self-reports (N = 4 of 70). The references against which the sensor data were compared were a metrological device and a medical examination in equal parts and, to a lesser extent, a third-party portable medical device. Finally, studies using machine learning as a validation method have become more numerous since 2010, at 17% of included studies.

## 4. Discussion

Gait monitoring of patients during daily life using commercial wearable sensors is a growing field and offers novel opportunities for future public health research. However, despite their rapid expansion, the use of commercial wearable sensors remains contested in the medical community: objections concern the quality of the data collected as well as the reliability of the technologies in a clinical context where the pathologies are diverse and sometimes combined [104]. Previous literature reviews on the validation of wearable sensors were interested in monitoring activity on healthy subjects [15,20,21,22] or have often placed a focus on a very specific medical application [18,23,24]. No review to date has focused on studies using wearable devices in a very general way to automatically detect gait in patients in their daily life and via machine learning, which is an approach increasingly used to learn a recognition task from data. By examining the validation methods and performances of wearable devices and sensors that automatically monitor patient gait, several major trends and challenges can be identified.

### 4.1. Trends and Challenges

**Acquisition context**. Most of the first studies were restricted to the laboratory environment and over short acquisition times (of the order of a few minutes). The first papers to report sensor validation in a free living environment were in 2011 [53,74]. As seen in Table 9, from 2017, studies of this type become more frequent [46,50,51,52,55,59,62,66,77,86,94,96,98,103] due to changes in the sensors, which are detailed in the following section.

**Sensors**. In this review, we observe that early research efforts attempted to find improvements for gait monitoring in patients by experimenting with new sensor types and/or sensor locations. The first paper to report the validation of a wearable sensor for monitoring gait in patients was in 2010 [90], but it did not become more prevalent until 2017, during which nine other papers on this subject were published [45,47,54,63,73,78,79,88,96]. Over time, research efforts have focused on refining validation protocols, whether in terms of the number of sensors or their locations, with emphasis on two major criteria: the ability of sensors to capture gait patterns and the practicality of everyday life. As seen in Table 2 and Table 3, the majority of studies (95%) used accelerometers and/or gyroscopes, typically embedded within an IMU or smartphone. This observation highlights the emergence of commercial wearable devices as a practical and user-friendly modality for gait monitoring in daily life. In addition to user adoption, commercial wearable devices also have engineering advantages, such as a compact format with suitable computing and power resources. If it is a single sensor, it is usually worn near the center of gravity, in a pocket [42,43,45,50,51,77,86], or on the chest [39,44,64,84,92] or pelvis [59,61,65,72,94,96].

Another trend that emerges from Table 2 is the fact that several sensors were used together and generally at various on-body locations [37,48,52,54,55,56,60,63,65,67,70,73,75,79,83,87,90,93,95,97,98,99,102]. However, using a multi-sensor system introduces several challenges, including the integration of different sampling rates and signal amplitudes, and how to align signals from multiple devices and, therefore, different clock times. Despite these challenges, the multi-sensor approach offers high potential for the real-time monitoring of gait, where multi-sensor fusion can provide context-awareness (e.g., if the patient stays mainly at home or leaves home from time to time) and can contribute to the optimization of power (e.g., a low-power sensor can trigger a higher-power sensor only when necessary).

**Ground truth**. Our review indicates that 53% of the included studies use annotations. As seen in Figure 5, there is still a strong reliance on annotations by groups of individuals (56%; mainly a group of patients versus a group of healthy subjects) followed by annotations made by experts on data from videos or measurements during the experiment (21%) and patient self-report (0.05%). These last two annotation methods are surely less numerous because they can be very costly and time-intensive and are also of questionable quality because maintaining logs is a process that is very burdensome to the participant and ultimately relies on their memory. This fact has namely led to the emergence of initiatives in terms of intelligent annotation [105].

Another trend in ground-truth validation is increasingly in favor of using a reference (46%) because of the confidence established from visually confirming the gait pattern being detected: this can be a metrological device (18%), a medical examination (20%), or a third-party portable medical device (8%). However, in this case, the data are not annotated and therefore do not allow for the use of conventional machine learning approaches. At best, the medical examination allows for a regression task to be carried out, which however, from a machine learning point of view, is more difficult. In general, comparisons are limited to traditional statistical tools such as correlations or difference tests [35,39,40,41,42,49,53,54,59,61,62,65,67,72,77,78,79,82,83,84,87,88,89,90,95,97,99,103].

**Machine learning**. The combination of machine learning algorithms and wearable sensors for gait analysis has shown promising results in validating the extraction of complex gait patterns [34,35,36,38,45,53,60,63,68,69,70,77,79,80,81,86,95,97,100].

As seen in Table 7, researchers have used machine learning on sensor data for different tasks: regression for continuous labelled data (speed, step length, or distance) [53,69,79,86] and classification of discrete labelled data such as groups of patients [35,36,38,45,68,80,86,97] or medical functional scores [34,45,63,95,100]. Classification, less commonly used for the validation of sensors, aims for higher-level analyses, namely to identify a robust methodology able to monitor patients in time while at the same time discriminating between a pathological and physiological gait, or the evolution of the disease studied on the basis of gait movements.

The types of machine learning algorithm families have evolved over time, with standard approaches being used before 2017 and the appearance of deep learning approaches with automatic feature extraction without human intervention for the first time in 2018 [77], which are unlike most traditional machine learning algorithms. It should be noted that, in the context of the papers studied in this review [60,70,77,80], these approaches concern studies with a significant number of patients (≥30) or/and relatively long acquisition times [77,80] in order to guarantee a sufficiently representative and realistic sample. Other studies based on machine learning preferred more standard approaches with a small number of expert features if their samples were more limited regarding the number of patients [38,63,68,69,79,81,86,95,100] or the acquisition time [34,35,36,45,97]. Comparing the results of the different studies in terms of performance seems, at this stage, to be a difficult task because, as stated previously, it depends on the complexity of the task to be performed and on the complexity of the machine learning algorithm implemented.

Finally, it should be mentioned that machine learning also has drawbacks, with the first being the computational time required to train a model [106]. This is justified for complex analysis tasks such as classification or significant performance increases for a regression task. Moreover, ML may require the adjustment of hyperparameters that may demand theoretical knowledge in optimization. Finally, ML tends to be more difficult to interpret for a clinician who looks for the most relevant parameters to analyze the gait patterns of patients. However, it should be noted that recent initiatives have been carried out to demystify these two points [107,108].

### 4.2. Recommendations

Advanced inertial sensors, including accelerometers and gyroscopes, are commonly integrated into smartphones and smart devices nowadays. Therefore, it is very convenient and cheap to collect inertial gait data to achieve gait monitoring with high accuracy. Most existing validation methods ask the person to walk along a specified road (e.g., a straight lounge) and/or at a normal speed. Obviously, such strict requirements heavily limit its wide application, which motivates us to give some recommendations for future work in this context.

**Data acquisition**. A first step would be to precisely define validation protocols—by consulting the medical staff—adapted to the study of chronic pathologies. Indeed, many studies only validate sensors for a given medical application without having tested them outside the laboratory, on a very limited number of patients, and over a relatively short time window (at most a few hours). The protocol to be defined should therefore impose experimentation constraints closer to the daily life of patients, namely the data should be acquired at home, on a sufficient number of patients, and over a sufficiently long acquisition period (several weeks or even months).

It would also be necessary to define within the protocol which types of sensors would be more suitable according to the studied pathology, how many sensors would be necessary, and where to place them on the patient [18]. There is a clear trade-off between the accuracy of the recorded data and the invasiveness of the portable system: the greater the number of sensors and the more varied they are placed on different parts of the patient’s body, the more accurate the measurements will be, but this is at the expense of a practical, accommodating, and portable use.

**Data collection and processing**. Today, most sensors record a lot of data about their users. However, most wearable devices do not have the memory and computing power to process and analyze all of the recorded signals. Faced with this problem, two solutions are generally considered: either the system uses only a part of the recorded data to provide accurate indicators (throwing away a massive amount of potentially interesting data) [109,110] or the system stores and analyzes all raw data on the cloud [111,112]. The latter option is often problematic because the traditional architecture is centralized and offers little protection against potential cyber attacks. Centralizing raw data on a server poses some risk, especially if the data is sent to an external server, as it facilitates access to malicious attackers. A more reliable and secure alternative regarding the collection and processing of data would therefore be to process the raw inertial signal on the user’s smartphone and to transfer only relevant features unlinked to the identity of users to the cloud [113,114]. Finally, the mobile clients associated with wearable devices have to send a lot of data to a centralized server for training and model inference. This is especially difficult due to user billing plans and user privacy. Thus, very recently, decentralized architectures dedicated to machine learning have emerged [115].

**Validation**. It is mandatory to ensure that sensor recordings are accurate and sensitive enough for medical diagnosis and prognosis. This is crucial to ensure not only the generalizability of a sensor within a target population but also its ability to measure day-to-day variability data, which can be corroborated with disease symptoms. To this end, data acquired by commercial wearable sensors should be systematically compared to data acquired by reference medical devices (i.e., reliable gold standard systems, medical scores, or groups of subjects). Machine learning approaches make it possible to loosen the strict framework of acquisition protocols but must ensure that the data set collected for training is large, labelled, and realistic. Deep approaches, which automatically select features from data, offer very interesting perspectives given that feature extraction is a task that can take teams of data scientists years to accomplish. It augments the powers of small expert teams, which by their nature do not scale.

**Statistical models versus ML**. Statistical models are designed for inference about the relationships between variables within the data and are designed for data with a few dozen input variables and small sample sizes. On the other hand, machine learning models are designed to make the most accurate predictions possible. Statistical models can make predictions, but predictive accuracy is not their strength. Indeed, no training and test sets are necessary. Furthermore, machine learning aims to build a model that can make repeatable predictions in a high-dimensional space without formulating a hypothesis on the underlying data generation mechanism. ML methods are particularly useful when the number of input variables exceeds the number of samples [116]. Hence, using machine learning in a validation task highly depends on the purpose of the study. To prove that a sensor is able to respond to a certain kind of stimuli (such as a walking speed), a statistical model should be used. Conversely, to predict from a collection of different sensors whether a patient is affected by a certain grade of a disease affecting the musculoskeletal system, machine learning is probably the best approach. Indeed, this multi-dimensional space (one or more for each sensor) is in fact difficult to interpret and therefore to analyze. The ML model would then probably be a neural network or a random forest in order to take into account the nonlinearities resulting from the complex relationship between the physical sensors and the classification output.

## 5. Conclusions

The field of gait monitoring in patients is still emerging, and the accuracy of commercial wearable sensors still depends on careful constraints during data acquisition. Collecting data in daily life is considerably more challenging than conducting research in a laboratory. In free-living conditions, continuous control of the sensors, participants, and hardware or software is lost. Therefore, successful sensor deployment requires really robust algorithms. If the objective is to be able to monitor the gait completely freely over a long period of time, precision must be valued. Considering this review of the last 10 years in the field, validation takes an increasingly important place in the literature, with the number of studies having gradually increased since 2010. In these studies, a significant part of the validation was based on traditional statistical approaches (75%) with a stable contribution of machine learning-based approaches (25%). Machine learning approaches are algorithms that should be considered for the future. These are in fact data-based approaches, which, as long as the data collected are numerous, annotated, and representative, allow for the training of an effective model. It should be noted that commercial wearable sensors allowing for increased data collection and good patient adherence through efforts of miniaturization, energy consumption, and comfort will contribute to its future success.

## Figures and Tables

**Table 1:** Frequency of studies according to conditions of data collection (laboratory or free living) and acquisition time *t* (from a few minutes to more than a year).

Acquisition Time	t < 1 h	1 ≤ t < 24 h	1 ≤ t < 7 d	1 ≤ t < 4 w	1 ≤ t < 12 m	t ≤ 1 y
Laboratory (N = 53)	46	3	0	1	2	1
Free Living (N = 17)	1	1	1	8	3	3

**Table 2:** Criteria related to commercial wearable devices through the 70 selected papers. Abbreviations used in the column “No. of device(s)”: IMU (Inertial Motion Unit), S (Sensor), and SPHN (Smartphone). Abbreviations used in the column “Sensor Type(s)”: A (accelerometer), G (gyroscope), M (magnetometer), and O (others).

Author	No. of Device(s)	Sensor Type(s)	Location of Device(s)	Sensor Model, Brand
Salarian et al. [90]	7 (IMU)	A,G	Forearms, shanks, thighs, sternum	Physilogs, BioAGM
Dobkin et al. [53]	2 (S)	A	Both ankles	GCDC, LLC
Kozey-Keadle et al. [74]	2 (S)	A	Right leg, right side of the hip	activPAL, PALF GT3X, ActiGraph
Munguía-Izquierdo et al. [82]	1 (IMU)	A,O	Arm	SenseWear, Bodymedia
Item-Glatthorn et al. [65]	5 (S)	A	Chest, thigh, forefoot	MiniSun, IDEEA
Grimpampi et al. [61]	1 (IMU)	A,G	Lumbar spine	Freesense, Sensorize
Schwenk et al. [92]	1 (IMU)	A,G	Chest	Physilog, GaitUp
Juen et al. [68]	1 (SPHN)	A	Pants pocket or fanny pack	Galaxy Ace, Samsung
Juen et al. [69]	2 (SPHN and S)	A	L3 vertebra	Galaxy Ace/4, Samsung
Sprint et al. [95]	3 (IMU)	A,G	Lumbar spine, shank	Shimmer3, Shimmer
Capela et al. [43]	1 (SPHN)	A,G,M	Rear pocket	Z10, BlackBerry
Schwenk et al. [93]	5 (IMU)	A,G,M	Shank, thigh, lower back	LegSys, BioSensic
Isho et al. [64]	1 (SPHN)	A	Torso	Xperia Ray SO-03C, Sony
Wuest et al. [102]	8 (IMU)	A,G	Wrists, shanks, trunk, feet, back	Physilog, GaitUp
Raknim et al. [86]	1 (SPHN)	A	Free (pocket, during phone call, on the bag during walk)	HTC and Samsung
Ferrari et al. [57]	2 (IMU)	A,G	Shoes	EXLs1 and EXLs3, EXEL
Brinkløv et al. [42]	1 (SPHN)	A	Pants pocket, jacket pocket	Iphone 5C, Apple
El-Gohary et al. [54]	3 (IMU)	A,G	Lumbar vertebra, feet, ankles	Opal, APDM
Ilias et al. [63]	4 (IMU)	A,G	Upper, lower limbs, wrists, legs	Shimmer3, Shimmer
Maqbool et al. [78]	1 (IMU)	A,G	Shank	MPU 6050, InvenSense
Terrier et al. [96]	1 (S)	A	Right hip	wGT3X-BT, ActiGraph
Rogan et al. [88]	1 (IMU)	A,G	Lateral malleolus	RehaWatch, Hasomed
Chiu et al. [47]	1 (SPHN)	A	Shin	Zenfone 2, ASUS
Cheng et al. [45]	1 (SPHN)	A	Carried in fanny pack	Galaxy S5, Samsung Optimus Zone2, LG
Kobsar et al. [73]	4 (IMU)	A,G	Foot, shank, thigh, lower back	iNEMO, STmicroelectronics
McGinnis et al. [79]	5 (IMU)	A	Sacrum, thighs, shanks	BioStampRC, MC10
Lipsmeier et al. [77]	1 (SPHN)	A,G,M,O	Hand, trouser pocket, belt	Galaxy S3 mini, Samsung
Kleiner et al. [72]	1 (IMU)	A,G,M	L5 verterbra	BTS G-walk, BTS G-Sensor
Carpinella et al. [44]	1 (IMU)	A,G,M	Sternum	MTw, Xsens
Jayaraman et al. [67]	4 (S)	A,O	Arm, waist, ankle	wGT3X-BT, ActiGraph Metria-IH1, Vandrico
Jang et al. [66]	1 (IMU)	A,O	Wrist	Mi band 2, Xiaomi
Derungs et al. [52]	6 (IMU)	A,G,M	Wrists, arms, thighs	Shimmer3, Shimmer
Mileti et al. [81]	10 (IMU and S)	A,G,M,O	Feet	Mtw, MTw, Xsens
Aich et al. [35]	2 (S)	A	Knees	Fit Meter, Fit.Life
Cheong et al. [46]	1 (IMU)	A	Wrists	Urban S, Partron Co
Ata et al. [40]	2 (SPHN and S)	A	Hand, hip	iPhones SE/6/7/7+, Apple GT9X, ActiGraph
Kim et al. [70]	3 (SPHN)	A,G	Waist, pocket, ankle	Nexus 5, Google
Vadnerkar et al. [100]	1 (IMU)	A,G	Feet	Shimmer 2r, Shimmer
Rosario et al. [51]	1 (SPHN)	A,G	Trouser pocket	Galaxy S3, Samsung
Lemoyne et al. [76]	1 (SPHN)	A	Malleolus	iPhone, Apple
Dasmahapatra et al. [50]	1 (S)	A	Belt, pocket, or bra	Fitbit One, Fitbit
Schliessmann et al. [91]	2 (IMU)	A,G,M	Feet	RehaGait, HASOMED GmbH
Ummels et al. [99]	9 (IMU and S)	other	Leg, belt, wrist	UP24, Jawbone Lumoback, Lumo Bodytech Moves, ProtoGeo Oy Accupedo, Corusen LLC Walking Style X, Omron
Banky et al. [41]	1 (SPHN)	G		Galaxy S5, Samsung
Flachenecker et al. [58]	2 (IMU)	A,G	Shoes	Shimmer 3, Shimmer
Gadaleta et al. [60]	3 (IMU)	A,G,M	L5 lumbar vertebrae, ankles	Opal, APDM
Teufl et al. [97]	7 (IMU)	A,G	Pelvis, both foot, both thighs	MTw Awinda, Xsens
Angelini et al. [37]	3 (IMU)	A,G	L5 lumbar vertebra, ankles	MTw Xsens Opal, APDM
Antos et al. [38]	2 (S and SPHN)	A,G	Waist, wrist	Nexus 5, Google wGT3X-BT, Actigraph
Compagnat et al. [48]	9 (S)	A,O	Wrists, ankles, hip, arm, neck	GT3x, Actigraph Sensewear, Body Media
Newman et al. [84]	1 (IMU)	A,G	Interclavicular notch	Opal, APDM
Ullrich et al. [98]	3 IMU	A,G	Ankles, shoes	Shimmer2R, Shimmer
Wang et al. [101]	2 (IMU)	A,G	Pectoralis major	BioStampRC, MC10
Pavon et al. [85]	2 (S)	A	Ankle	GT3x+, ActiGraph
Arcuria et al. [39]	1 (SPHN)	A	Breastbone	Galaxy J3, Samsung
Erb et al. [55]	7 to 16 (IMU)	A,G,M,O	Wrists, torso, thigh, feet	Shimmer, Shimmer
Aich et al. [36]	2 (S)	A	Knees	Fit Meter, Fit. Life
Rubin et al. [89]	1 (SPHN)	A,G	Pants pocket, belt	iPhone 6, Apple
Henriksen et al. [62]	1 (IMU)	A,O	Wrist	M430 AT, Polar
Shema-Shiratzky et al. [94]	1 (IMU)	A	Lower Back	Opal, APDM and AX3, Axivity
Abdollahi et al. [34]	1 (IMU)	A,G	Sternum	9DOF Razor IMU, Sparkfun
Kim et al. [71]	2 (IMU)	A,G	Shoe, ankle	GT9X Link, ActiGraph
Lemay et al. [75]	5 (IMU)	A,G,O	Feet, shanks, sacrum	Physilog, GaitUp
Meisel et al. [80]	1 (S)	A,O	Wrist or ankle	E4, Empatica
Fantozzi et al. [56]	5 (IMU)	A,G,M	Trunk, pelvis, thigh, shank, foot	Opal, APDM
Zhai et al. [103]	2 (SPHN and S)	A	Wrist, pocket	Galaxy S4 mini, Samsung GT3X+, ActiGraph

Revi et al. [87]	3 (IMU)	A	Shank, thigh, pelvis	MTw Awinda, Xsens
Compagnat et al. [49]	2 (S)	A	Non-paretic hip	GT3x, ActiGraph
Furtado et al. [59]	1 (S)	A	L5 lumbar vertebrae within the pocket of a belt	AX3, Axivity
Na et al. [83]	5 (IMU)	A,G	Femur, tibia, pelvis, sacral ridge	3D Myomotion, Noraxon

**Table 3:** Frequency of devices and sensor types in included studies. The device is the tracker used by the patient (first column), which may include different sensors that are detailed in the second column. Note that, since a device can use several sensors, the total number of occurrences in the second column is much greater than that of the first column.

Device Type		Sensor Type	
IMU	39	Accelerometer	39 (100%)
		Gyroscope	30 (77%)
		Magnetometer	8 (20%)
		Others	7 (18%)
Sensors	17	Accelerometer	14 (82%)
		Gyroscope	1 (0.7%)
		Magnetometer	1 (0.7%)
		Others	4 (3%)
Smartphones	18	Accelerometer	17 (94%)
		Gyroscope	7 (38%)
		Magnetometer	2 (11%)
		Others	1 (5%)

**Table 4:** Frequency of sensor locations reported on the patient from the included studies. These different locations were classified into the four categories described in Section 2.6.

Superior	Inferior	Chest	Free
12	42	34	12

**Table 5:** Frequency of features extracted from sensor signal reported from the included studies. These different features were classified into the three categories described in Section 2.6.

Low Level		Medium Level		High Level	
Total	6	Total	20	Total	49
		Magnitude mean	11	Step length	20
		Magnitude standard deviation	10	Number of steps	18
		Peak frequency	9	Cadence	15
		Mean crossing rate	5	Speed	11

**Table 6:** Evaluation criteria through the 70 selected papers. Abbreviations used in the column “Evaluation method”: stats (descriptive statistics), stats + test (descriptive statistics + statistical tests), LM + test (linear models + statistical tests), ML (machine learning), and ML+test (machine learning + statistical tests). Abbreviations used in the column “Evaluation outcomes”: *r* (correlation coefficient), $R^{2}$ (coefficient of determination), ICC (intraclass correlation coefficient), AUC (area under curve, sen (sensitivity), spe (specificity), IQR (interquartile range), FN (false negatives), FP (false positives), and acc (accuracy).

Author	Ground-Truth Method	Gait Descriptors	# of Descriptors	Evaluation Method	Evaluation Outcomes
Salarian et al. [90]	controls, medical	high	20	stats + test	*p*-value < 0.023
Dobkin et al. [53]	controls, metrologic	medium	8	ML + test	*r* = 0.98
Kozey-Keadle et al. [74]	expert	high	3	stats	$R^{2}$ = 0.94
Munguía-Izquierdo et al. [82]	med device	high	1	stats + test	*r* = 0.87–0.99
Item-Glatthorn et al. [65]	metrologic	high	6	stats + test	ICC = 0.815–0.997
Grimpampi et al. [61]	metrologic	low, medium	3	stats + test	*r* = 0.74–0.87
Schwenk et al. [92]	controls, user	high	9	stats + test	AUC = 0.77, sen/spe = 72%/76%
Juen et al. [68]	medical	medium	8	ML	acc = 89.22–94.13%
Juen et al. [69]	med device	medium	9	ML	error < 10.2%
Sprint et al. [95]	medical	medium,high	18	ML + test	*r* = 0.97
Capela et al. [43]	expert	high	10	stats	time difference = 0.014 s
Schwenk et al. [93]	controls, user	high	6	LM + test	*p*-value < 0.022
Isho et al. [64]	controls, user	medium	3	ML + test	AUC = 0.745
Wuest et al. [102]	controls, medical	high	13	stats + test	*p*-value < 0.02
Raknim et al. [86]	controls	high	2	ML	acc = 94%
Ferrari et al. [57]	metrologic	high	4	LM + test	error = 2.9%
Brinkløv et al. [42]	med device	medium	6	LM + test	$R^{2}$ = 0.45–0.60
El-Gohary et al. [54]	metrologic, controls	high	7	stats + test	*r* = 0.592–0.992
Ilias et al. [63]	expert	medium	152	ML + test	*r* = 0.78–0.79
Maqbool et al. [78]	metrologic, controls	high	1	stats	time difference = 50 ms
Terrier et al. [96]	controls, medical	high	4	LM + stats	$R^{2}$ = 0.44
Rogan et al. [88]	metrologic	high	6	stats + test	*p*-value < 0.05
Chiu et al. [47]	controls	medium	1	stats + test	*p*-value < 0.027
Cheng et al. [45]	med device, medical	medium,high	10	ML	NA
Kobsar et al. [73]	medical	medium	38	LM + test	acc = 74–81.7%
McGinnis et al. [79]	metrologic, controls	medium	32	ML + test	speed difference = 0.12–0.16 m/s
Lipsmeier et al. [77]	controls, medical	high	6	ML + test	*p*-value < 0.055
Kleiner et al. [72]	metrologic, medical	high	1	stats	time difference = 0.585 s
Carpinella et al. [44]	medical, controls	high	5	stats + test	*r* = −0.367–0.536
Jayaraman et al. [67]	expert, metrologic	high	3	stats + test	*p*-value < 0.05
Jang et al. [66]	controls	high	5	stats + test	*p*-value < 0.02
Derungs et al. [52]	expert	medium	8	LM + test	sen/spe = 80%/94%
Mileti et al. [81]	controls, medical	low	3	ML + test	AUC = 0.48–0.98
Aich et al. [35]	metrologic, controls	high	28	ML	acc = 88%
Cheong et al. [46]	controls	high	1	stats + test	*p*-value < 0.04
Ata et al. [40]	expert, med device	high	3	stats	$R^{2}$ = 0.9–0.92
Kim et al. [70]	expert	medium	8	ML	sen/spe = 93.8%/90.1%
Vadnerkar et al. [100]	expert	low	1	LM + test	acc = 84%, sen/spe = 75.9%/95.9%
Rosario et al. [51]	controls, medical	high	2	stats + test	*r* = 0.472
Lemoyne et al. [76]	controls	high	5	stats + test	*p*-value < 0.05
Dasmahapatra et al. [50]	controls, medical	high	6	LM + test	*p*-value < 0.05
Schliessmann et al. [91]	controls	high	4	stats + test	*p*-value < 0.05
Ummels et al. [99]	metrologic	high	1	stats + test	*r* = −0.02–0.33
Banky et al. [41]	metrologic, controls	low	3	stats + test	*r*=0.8
Flachenecker et al. [58]	controls, medical	high	8	stats + test	*r* = −0.583–0.668
Gadaleta et al. [60]	metrologic	low	24	ML	bias = −0.012–0.000, IQR = 0.004–0.032
Teufl et al. [97]	metrologic, controls	high	10	ML + test	acc = 0.87–0.97
Angelini et al. [37]	expert, controls	high	14	stats + test	*p*-value < 0.05
Antos et al. [38]	expert, controls	medium	56	ML + test	acc = 0.90–0.95
Compagnat et al. [48]	expert	high	2	stats + test	*p*-value < 0.05
Newman et al. [84]	controls, medical	high	9	stats + test	*p*-value < 0.05
Ullrich et al. [98]	expert	medium	7	stats + test	sen/spe = 98%/96%
Wang et al. [101]	controls	medium	1	stats + test	*p*-value < 0.05
Pavon et al. [85]	controls, medical	high	3	stats + test	*p*-value < 0.16
Arcuria et al. [39]	metrologic, controls, medical	high	1	stats + test	*r* = −0.72–0.91
Erb et al. [55]	user, expert	high	2	stats + test	FN = 35%, FP = 15%
Aich et al. [36]	metrologic, controls, medical	high	5	ML	acc = 88.46%
Rubin et al. [89]	med device	high	1	stats + test	$R^{2}$ = 0.72
Henriksen et al. [62]	med device	high	4	stats	*r* = 0.446–0.925
Shema-Shiratzky et al. [94]	controls, expert	high	5	stats + test	*p*-value < 0.05
Abdollahi et al. [34]	medical	medium	920	ML	acc = 60–75%
Kim et al. [71]	controls	high	5	stats + test	*p* < 0.05
Lemay et al. [75]	medical, controls	high	6	LM + test	*r* = −0.49–0.498
Meisel et al. [80]	expert	low	6	ML + test	acc = 43%
Fantozzi et al. [56]	controls	high	14	LM + test	NA
Zhai et al. [103]	med device, controls, medical	medium	14	stats + test	*r* = 0.43–0.605
Revi et al. [87]	metrologic	high	8	stats	$R^{2}$ = 0.90–0.93
Compagnat et al. [49]	med device	high	1	stats + test	*r* = 0.44–0.87
Furtado et al. [59]	metrologic, controls, medical	medium,high	10	stats + test	*p*-value < 0.024
Na et al. [83]	metrologic, controls	high	6	stats + test	*p*-value < 0.04

**Table 7:** Selection of papers that use machine learning methods in validation. Abbreviations used in the column “Model type”: SVM (support vector machine), GPR (gaussian process regression), NN (neural network), RF (random forest), LSTM (long short time memory), HMM (hidden markov model), kNN (k-nearest neighbors), CNN (convolutional neural network), ROC (receiver operating characteristic), and LDA (linear discriminant analysis). Abbreviations used in the column “Outcome”: r (correlation coefficient), NRMSE (normalized root mean square error), RMSE (root mean square error), AUC (area under curve), sens (sensitivity), spe (specificity), and IQR (interquartile range). Studies that use raw data as input have a number of descriptors that correspond to the number of sensors and/or axes multiplied by the length of the recorded data. This is noted (*n) in the table.

Author	Task	Model Type	Training Size	# of Descriptors	Outcome
Dobkin et al. [53]	Speed prediction	Naive Bayes	NA	24	*r* = 0.98
Juen et al. [68]	Healthy/patient	SVM	10–20	8	accuracy = 89.22–94.13%
Juen et al. [69]	Speed prediction Distance prediction	GPR NN SVM	24	60	error rate = 2.51% error rate = 10.2%
Sprint et al. [95]	FIM motor score prediction	SVM RF	19	18	NRMSE = 10–30%
Raknim et al. [86]	Step length estimation Before/after PD	SVM	1	2	accuracy = 98% accuracy = 94%
Ilias et al. [63]	Motor function prediction	SVM	6	152	RMSE = 0.46-0.70 *r* = 0.78–0.79
Cheng et al. [45]	3 pulmonary severity stages	SVM	22–25	10	NA
McGinnis et al. [79]	Walking speed	SVM	16	32	RMSE = 10–20%
Lipsmeier et al. [77]	Activities	LSTM	44	6 (*n)	accuracy = 98%
Mileti et al. [81]	4 gait phases	HMM	1–11	3 (*n)	AUC = 0.48–0.98 sens= 80–100% spe = 70–90%
goodness Index = 10–40%
Aich et al. [35]	Healthy/patient	SVM Decision tree Naive Bayes kNN	36	28	accuracy=91.42% sens/spe = 90.9%/91.2%
Kim et al. [70]	Walking/freezing	CNN	29	8 (*n)	f1-score = 91.8 sen/spe = 93.8%/90.1%
Vadnerkar et al. [100]	Gait quality	ROC decision boundary	8	1	accuracy = 84% sen/spe = 75.9%/95.9%
Gadaleta et al. [60]	Right/left foot events	CNN	138	24 (*n)	bias = −0.012–0.000 IQR = 0.004–0.032
Teufl et al. [97]	Healthy/patient	SVM	40	10	accuracy = 87–97%
Antos et al. [38]	With/without assistance	RF SVM Naive Bayes Logistic regression LDA	1–13	56	accuracy = 90–95%
Aich et al. [36]	Healthy/patient	kNN SVM Naive Bayes Decision tree	62	10	accuracy = 88.5% sens/spe = 92.9%/90.9%
Abdollahi et al. [34]	Risk of disability	SVM Perceptron	93	920	accuracy = 60–75%
Meisel et al. [80]	Seizure/healthy	LSTM	68	6 (*n)	accuracy = 43%

**Table 8:** Frequency of studies using less than 10 descriptors, between 10 and 100 descriptors and more than 100 descriptors for the validation of both statistical and ML methods.

Number of Studies	<10	10–100	>100
Statistical	43	8	0
ML	3	9	7

**Table 9:** Data acquisition criteria through the 70 selected papers. Abbreviations used in the column “Duration of data collection”: min (t <1 h), hours (1 ≤ t < 24 h), days (1 ≤t< 7 days), weeks (1 ≤ t < 4 weeks), months (1 ≤ t < 12 months), and year (t ≥ 1 year). Finally, the cohort size is given as the number of patients.

Author	Year	Pathology	Cohort Size	Duration of Data Collection	Condition Data Collection
Salarian et al. [90]	2010	Parkinson	12	min	Laboratory
Dobkin et al. [53]	2011	Stroke	12	min (Lab), days (FL)	Both
Kozey-Keadle et al. [74]	2011	Obesity	20	hours	Free living
Munguía-Izquierdo et al. [82]	2012	Fibromyalgia	25	min	Laboratory
Item-Glatthorn et al. [65]	2012	Osteoarthritis	26	min	Laboratory
Grimpampi et al. [61]	2013	Hemiplegia/Parkinson	24	min	Laboratory
Schwenk et al. [92]	2014	Dementia	77	days	Free living
Juen et al. [68]	2014	Lung disease	30	min	Laboratory
Juen et al. [69]	2014	Lung disease	25	min	Laboratory
Sprint et al. [95]	2015	Diverse	20	min	Laboratory
Capela et al. [43]	2015	Lung disease	15	min	laboratory
Schwenk et al. [93]	2016	Cancer	22	hours	laboratory
Isho et al. [64]	2015	Stroke	24	min	Laboratory
Wuest et al. [102]	2016	Stroke	26	min	Laboratory
Raknim et al. [86]	2016	Parkinson	1	years	Free living
Ferrari et al. [57]	2016	Parkinson	14	min	Laboratory
Brinkløv et al. [42]	2016	Diabete	27	min	Laboratory
El-Gohary et al. [54]	2017	Multiple sclerosis	52	min	Laboratory
Ilias et al. [63]	2017	Parkinson	19	min	Laboratory
Maqbool et al. [78]	2017	Amputee	2	min	Laboratory
Terrier et al. [96]	2017	Chronic Pain	66	weeks	Both
Rogan et al. [88]	2017	Old Age	23	min	Laboratory
Chiu et al. [47]	2017	Ankle instability	15	min	Laboratory
Cheng et al. [45]	2017	Cardiopulmonary disease	25	min	Laboratory
Kobsar et al. [73]	2017	Osteoarthritis	39	months	Laboratory
McGinnis et al. [79]	2017	Multiple sclerosis	30	min	Laboratory
Lipsmeier et al. [77]	2018	Parkinson	44	months	Free living
Kleiner et al. [72]	2018	Parkinson	30	min	Laboratory
Carpinella et al. [44]	2018	Diverse	30	min	Laboratory
Jayaraman et al. [67]	2018	Spinal Cord Injury	18	hours	Laboratory
Jang et al. [66]	2018	Old Age	22	years	Free living
Derungs et al. [52]	2018	Hemiparesis	11	weeks	Free living
Mileti et al. [81]	2018	Parkinson	26	min	Laboratory
Aich et al. [35]	2018	Parkinson	51	min	Laboratory
Cheong et al. [46]	2018	Cancer	102	months	Free living
Ata et al. [40]	2018	Artery disease	114	min	Laboratory
Kim et al. [70]	2018	Parkinson	32	min	Laboratory
Vadnerkar et al. [100]	2018	Old Age	16	min	Laboratory
Rosario et al. [51]	2018	Cardiac disease	66	months	Free living
Lemoyne et al. [76]	2018	Hemiplegia	1	min	Laboratory
Dasmahapatra et al. [50]	2018	Multiple Sclerosis	114	weeks	Free living
Schliessmann et al. [91]	2018	Diverse	41	min	Laboratory
Ummels et al. [99]	2018	Diverse	130	years	Laboratory
Banky et al. [41]	2019	Diverse	35	hours	Laboratory
Flachenecker et al. [58]	2019	Multiple sclerosis	102	min	Laboratory
Gadaleta et al. [60]	2019	Parkinson	71	min	Laboratory
Teufl et al. [97]	2019	Arthroplasty	20	min	Laboratory
Angelini et al. [37]	2019	Multiple sclerosis	26	min	Laboratory
Antos et al. [38]	2019	Old Age	20	min	Laboratory
Compagnat et al. [48]	2019	Stroke	35	min	Laboratory
Newman et al. [84]	2020	Brain injury	12	min	Laboratory
Ullrich et al. [98]	2020	Parkinson	128	min	Both
Wang et al. [101]	2020	Post Sternotomy	22	min	Laboratory
Pavon et al. [85]	2020	Disability	46	days	Laboratory
Arcuria et al. [39]	2020	Cerebellar ataxia	40	min	Laboratory
Erb et al. [55]	2020	Parkinson	34	weeks	Free Living
Aich et al. [36]	2020	Parkinson	48	min	Laboratory
Rubin et al. [89]	2020	Diverse	78	min	Laboratory
Henriksen et al. [62]	2020	Obesity	16	years	Free living
Shema-Shiratzky et al. [94]	2020	Multiple Sclerosis	44	min	Both
Abdollahi et al. [34]	2020	Chronic pain	94	min	Laboratory
Kim et al. [71]	2020	Amputation	17	min	Laboratory
Lemay et al. [75]	2020	Spinal cord injury	18	min	Laboratory
Meisel et al. [80]	2020	Epilepsy	69	months	Laboratory
Fantozzi et al. [56]	2020	Old Age	9	min	Laboratory
Zhai et al. [103]	2020	Multiple Sclerosis	67	min (Lab), weeks (FL)	Both
Revi et al. [87]	2020	Stroke	5	min	Laboratory
Compagnat et al. [49]	2020	Stroke	26	min	Laboratory
Furtado et al. [59]	2020	Amputation	34	hours (Lab), weeks (FL)	Both
Na et al. [83]	2020	Osteoarthritis	39	min	Laboratory

## Data Availability

Data is contained within the article.

## Abbreviations

The following abbreviations are used in this manuscript:

6MWT	Six-minute walk test
ML	Machine learning
SD	Standard deviation
IMU	Inertial Measurement Unit

## Appendix A. Extraction from Databases

**Table A1:** Search term strategy.

Database	Search String	Records
ACM	[[Abstract: gait] OR [Abstract: actimetry] OR [Abstract: actigraphy]	17
	OR [Abstract: walk]] AND [[[Abstract: smartphone] OR [Abstract: wearable]	
	OR [Abstract: iot]] AND [[Abstract: “chronic disease”] OR [Abstract: rehabilitation]	
	OR [Abstract: medicine]] AND [[Abstract: validity] OR [Abstract: reliability]	
	OR [Abstract: reproductibility or validation] OR [Publication Title: gait]	
	OR [Publication Title: actimetry] OR [Publication Title: actigraphy]	
	OR [Publication Title: walk]] AND [[Publication Title: smartphone]	
	OR [Publication Title: wearable] OR [Publication Title: iot]	
	AND [Publication Title: “chronic disease”] OR [Publication Title: rehabilitation]	
	OR [Publication Title: medicine]] AND [[Publication Title: validity]	
	OR [Publication Title: reliability] OR [Publication Title: reproductibility or validation]]	
	AND [Publication Date: (01 January 2010 TO 31 October 2020)]	
Cochrane	((gait OR actimetry OR actigraphy OR walk) AND (smartphone OR wearable OR iot) AND	15
	(“chronic disease” OR rehabilitation OR medicine) AND (validity OR reliability OR	
	reproductibility OR validation)) in Title Abstract Keyword—between Jan 2010 and October 2020	
DBLB	(gait | walk | actimetry) (smartphone | device | iot) (valid | rehabilitation)	31
IEEE Xplore	((gait OR actimetry OR actigraphy OR walk) AND (smartphone OR wearable OR iot)	54
	AND (“chronic disease” OR rehabilitation OR medicine) AND (validity	
	OR reliability OR reproductibility or validation))	
PubMed	((gait OR actimetry OR actigraphy OR walk)	52
	AND (smartphone OR wearable OR iot) AND	
	(“chronic disease” OR rehabilitation OR medicine) AND	
	(validity OR reliability OR reproductibility or validation))	
	Filters: from 2010–2020	
Scholar	title:(gait smartphone “wearable device” rehabilitation validity)	1010
ScienceDirect	((gait OR actimetry) AND (smartphone OR iot) AND	3
#1	(“chronic disease” OR medicine) AND	
	(validity OR validation))	
ScienceDirect	((gait OR walk) AND (smartphone OR wearable) AND	10
#2	(rehabilitation OR medicine) AND	
	(validity OR reliability))	
ScienceDirect	((gait OR walk) AND (smartphone OR iot) AND	1
#3	AND (“chronic disease” OR medicine) AND	
	(validity OR validation))	
ScienceDirect	((gait OR walk) AND (smartphone OR wearable) AND	16
#4	AND (rehabilitation OR medicine) AND	
	(validity OR validation))	
ScienceDirect	((gait OR actimetry OR walk) AND	12
#5	(smartphone OR wearable OR iot) AND	
	rehabilitation AND validation)	
SCOPUS	TITLE-ABS-KEY((( gait OR actimetry OR actigraphy OR walk )	155
	AND ( smartphone OR wearable OR iot ) AND	
	( “chronic disease” OR rehabilitation OR medicine ) AND	
	( validity OR reliability OR reproductibility OR validation)))	
	AND PUBYEAR ≥ 2010 AND PUBYEAR ≤ 2020	
Web of Science	(TS = ((gait OR actimetry OR actigraphy OR walk)	148
	AND (smartphone OR wearable OR iot) AND (“chronic disease” OR	
	rehabilitation OR medicine) AND (validity OR reliability OR	
	reproductibility OR validation))) AND LANGUAGE: (English)	
	AND DOCUMENT TYPES: (Article) Indexes=SCI-EXPANDED,	
	SSCI, A&HCI, CPCI-S, CPCI-SSH, ESCI,	
	CCR-EXPANDED, IC Timespan=2010-2020	
